# Gene amplification-driven lncRNA SNHG6 promotes tumorigenesis via epigenetically suppressing p27 expression and regulating cell cycle in non–small cell lung cancer

**DOI:** 10.1038/s41420-022-01276-y

**Published:** 2022-12-09

**Authors:** Qi Wang, Wei Zhang, Dandan Yin, Zaibin Tang, Erbao Zhang, Weibing Wu

**Affiliations:** 1 Department of Thoracic Surgery, The First Affiliated Hospital of Nanjing Medical University, Nanjing, China; 2 Clinical Research Center, The Second Hospital of Nanjing, Nanjing University of Chinese Medicine, Zhong Fu Road, Gulou District, Nanjing, Jiangsu 210003 PR China; 3 Department of Epidemiology, Center for Global Health, School of Public Health, Nanjing Medical University, Nanjing, China; 4 Jiangsu Key Lab of Cancer Biomarkers, Prevention and Treatment, Jiangsu Collaborative Innovation Center for Cancer Personalized Medicine, Nanjing Medical University, Nanjing, China

**Keywords:** Non-small-cell lung cancer, Long non-coding RNAs

## Abstract

Long non-coding RNAs (lncRNAs) have been validated to play essential roles in non-small cell lung carcinoma (NSCLC) progression. In this study, through systematically screening GSE33532 and GSE29249 from Gene Expression Omnibus (GEO) database and bioinformatics analysis, we found the significant upregulation of SNHG6 in NSCLC. The activation of SNHG6 was driven by copy number amplification and high expression of SNHG6 indicated a poor prognosis. Functionally, the knockdown of SNHG6 inhibited NSCLC cell proliferation, migration, and suppressed the G1/S transition of the cell cycle. SNHG6 overexpression had the opposite effects. Mechanically, SNHG6 recruited EZH2 to the promoter region of p27 and increased H3K27me3 enrichment, thus epigenetically repressing the expression of p27, regulating the cell cycle, and promoting tumorigenesis of NSCLC. SNHG6 silencing restrained tumor growth in vivo and suppressed the expressions of cell cycle-related proteins in the G1/S transition. In conclusion, our study uncovered a novel mechanism of SNHG6 activation and its function. SNHG6 can be considered a potential target for the diagnosis and treatment of NSCLC in the future.

## Introduction

Lung cancer is one of the major malignant tumors that threaten global health, and its mortality ranks first of all malignancies while incidence ranks second-highest currently [1, 2]. Lung cancer can be divided into several categories based on histological subtypes, including small cell lung cancer (SCLC) and non-small cell lung carcinoma (NSCLC), and NSCLC accounts for the main part [3]. Despite the rapid advances in diagnosis and therapy, the prognosis of NSCLC is still unfavorable [4, 5]. Therefore, it is significant to explore new molecular targets to improve the treatment of NSCLC patients.

Long non-coding RNAs (lncRNAs), which were thought to be transcriptional noises previously, were a group of RNAs that lacked protein-coding abilities [6]. It revealed that lncRNAs participated in the progression of various tumors through diverse mechanisms, including interacting with miRNA, binding to RNA-binding protein (RBP), etc. [7–10]. In this study, by systematic analysis and screening for the differential-expressed lncRNAs in GSE33532 and GSE29249 of GEO, we identified small nucleolar RNA host gene 6 (SNHG6).

SNHG6 was a lncRNA that has been validated to serve as an oncogene in many malignancies. For example, SNHG6 interacted with hnRNPA1 by regulating alternative splicing of PKM in colorectal cancer [11]. SNHG6 was upregulated in primary breast cancers and promoted cell cycle progression [12]. SNHG6 sponged let-7c-5p to upregulate c-Myc in hepatocellular carcinoma [13]. In addition, SNHG6 served as a promising biomarker in the prediction of prognosis and clinicopathological features in varieties of human cancers [14]. However, the functional role and underlying mechanisms of SNHG6 have not been deeply understood in NSCLC. Especially, the activation mechanism of SNHG6 in tumorigenesis is unclear.

In this study, we first found that the upregulation of SNHG6 was partly mediated by the copy number amplification in NSCLC. Following, functional assays and animal experiments revealed that SNHG6 promoted NSCLC progression in vivo and in vitro. In terms of mechanism, SNHG6 epigenetically silenced p27 via recruiting EZH2 to its promoter region and increasing H3K27me3 enrichment. All these findings provided us with a deeper insight into the role of SNHG6 in NSCLC progression.

## Results

### Gene amplification-driven SNHG6 was aberrantly upregulated in NSCLC, which predicated a poorer prognosis

**Fig. 1:**
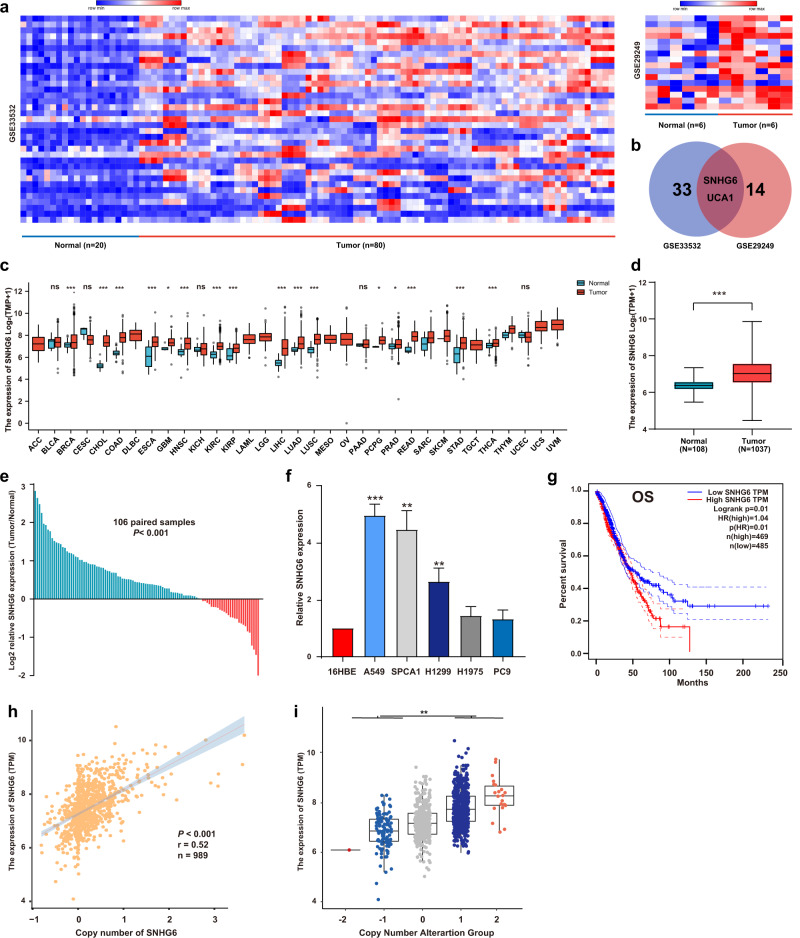
Gene amplification-driven SNHG6 was significantly upregulated in NSCLC and predicated a poor prognosis. **a** 35 lncRNAs were found upregulated in NSCLC tumor tissues from GSE33532 (20 normals vs 80 tumors) and 16 lncRNAs were found upregulated in NSCLC tumor tissues from GSE29249 (6 normals vs 6 tumors); **b** UCA1 and SNHG6 were identified in both datasets; **c** SNHG6 was upregulated in numerous malignant tumors in the TCGA database; **d** SNHG6 was upregulated in NSCLC tumor tissues compared with normal tissues in unpaired samples (Normal = 108, Tumor = 1037); **e** The differential expression level of SNHG6 in 106 paired samples (paired student’s *t*-test); **f** SNHG6 was upregulated in NSCLC cell lines (A549, SPCA1, and H1299) compared with 16HBE (unpaired student’s *t*-test); **g** High expression of SNHG6 was associated with a poor prognosis (OS, overall survival, log-rank test); **h** SNHG6 expression was positively related to the copy number of SNHG6 in NSCLC (*P* < 0.001, *r* = 0.52, *n* = 989); **i** SNHG6 expression in different copy number alteration group. Variables were presented as the mean ± SD (Standard deviation). ***P < 0.001, **P < 0.01.

Firstly, we screened the differential-expressed lncRNAs in the GEO database. As Fig. 1a showed, 35 lncRNAs were identified upregulated in NSCLC tumors from GSE33532 (20 normal tissues vs 80 normal tissues). Similarly, we observed 16 lncRNAs upregulated in tumor tissues from GSE29249 (6 normal tissues vs 6 tumor tissues). It demonstrated that SNHG6 and UCA1 were the intersections of high-expressed genes in the two datasets (Fig. 1b). Mounting evidence has revealed that UCA1 (urothelial cancer associated 1, UCA1) exerted crucial effects on NSCLC progression [15–18]. Nevertheless, the roles of SNHG6 were largely unknown in the tumorigenesis and development of NSCLC.

Firstly, we detected the expression level of SNHG6 in pan-cancer based on the UCSC Xena database (https://xenabrowser.net/datapages/). As Fig. 1c showed, SNHG6 was significantly upregulated in most malignancies, such as breast invasive carcinoma (BRCA), cholangiocarcinoma (CHOL), colon adenocarcinoma (COAD), esophageal carcinoma (ESCA), etc. Especially, SNHG6 was notably upregulated in NSCLC tumor tissues as well (Fig. 1d, unpaired samples, Normal = 108 vs Tumor = 1037, *P* < 0.001; Fig. 1e, 106 paired samples, *P* < 0.001). In line with the tissue expression, SNHG6 was upregulated in NSCLC cell lines as well (A549, SPCA1, and H1299) compared with the human bronchial epithelial cell line (16HBE) (Fig. 1f). A549 and SPCA1 were the cell lines with the highest SNHG6 expression, and they were chosen for the subsequent experiments. Furthermore, the high expression of SNHG6 was significantly associated with a poorer prognosis of NSCLC patients (Fig. 1g, OS: Overall survival, Log-rank *P* = 0.01).

Additionally, we found that SNHG6 expression was positively correlated with its copy number in NSCLC (Fig. 1h, *P* < 0.001, *r* = 0.52, *n* = 989). The same result was displayed for different copy number alteration groups in Fig. 1i. The findings revealed that the aberrant upregulation of SNHG6 in NSCLC may partially be driven by the copy number amplification of SNHG6.

### SNHG6 promoted cell proliferation and migration of NSCLC

**Fig. 2:**
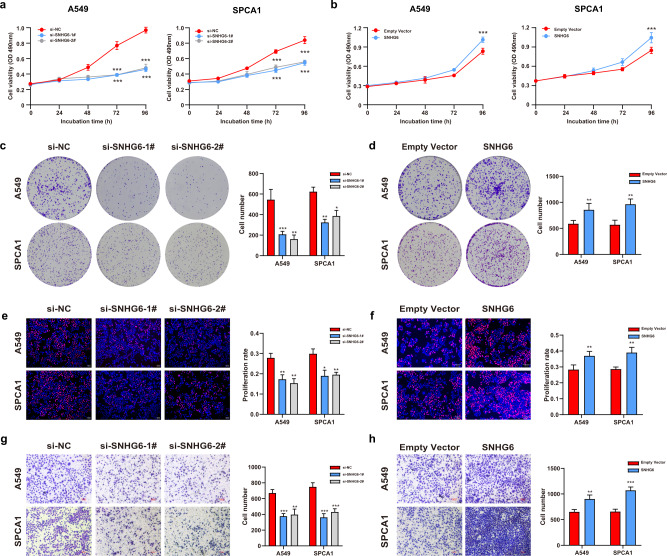
SNHG6 promoted cell proliferation and migration in NSCLC cell lines. **a** SNHG6 knockdown weakened the cell viability of A549 and SPCA1 cells; **b** SNHG6 overexpression increased cell viability of NSCLC cells; **c** SNHG6 inhibition decreased the formed colonies; **d** SNHG6 upregulation promoted cell proliferation according to colony-forming assays; **e**, **f** The cell proliferation rate of A549 and SPCA1 cells was evaluated using EdU assays after SNHG6 knockdown and overexpression; **g** SNHG6 silencing inhibited cell migration of A549 and SPCA1; **h** SNHG6 promoted cell migration of NSCLC cells. The results were from three independent experiments. Data were analyzed by unpaired student’s *t*-test. ***P < 0.001, **P < 0.01, *P < 0.05. Variables were presented as the mean ± SD.

To explore the biological function of SNHG6, we designed two small interfering RNAs (siRNAs) to knock down SNHG6 and constructed the overexpression plasmid to upregulate SNHG6 in NSCLC cells. As Figure S1a showed, the expression of SNHG6 was efficiently downregulated after transfection with si-SNHG6, and pcDNA3.1-SNHG6 upregulated SNHG6 expression as well (Fig. S1b). The MTT assays, colony formation assays, and EdU assays were carried out to evaluate cell proliferation. The cell viability of A549 and SPCA1 was inhibited after SNHG6 knockdown (Fig. 2a). Inversely, transfection with pcDNA3.1-SNHG6 significantly increased cell viability of NSCLC cells (Fig. 2b). The results of colony-forming assays were consistent with MTT assays (Fig. 2c, d). Similarly, SNHG6 silencing resulted in the reduction of cell proliferation rate according to the EdU assays (Fig. 2e), and SNHG6 overexpression produced results opposite to knockdown (Fig. 2f). Furthermore, transwell assays demonstrated that SNHG6 promoted cell migration while the silencing of SNHG6 suppressed the migratory abilities of A549 and SPCA1 cells (Fig. 2g, h). Taken together, the findings above suggested that SNHG6 could promote NSCLC progression in vitro.

### SNHG6 knockdown accelerated cell apoptosis and inhibited the G1/S transition of NSCLC cells

**Fig. 3:**
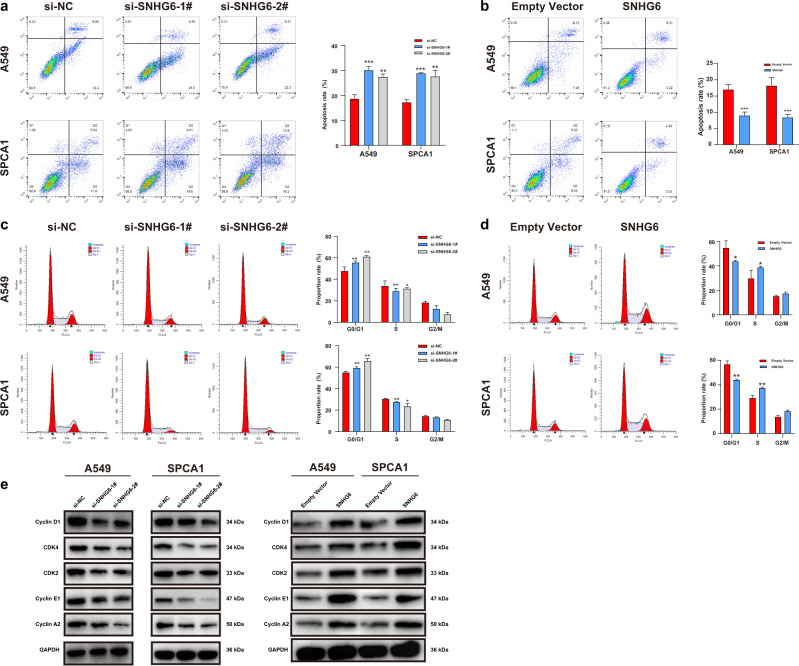
SNHG6 knockdown suppressed G1/S transition and promoted cell apoptosis. **a** SNHG6 knockdown facilitated cell apoptosis in NSCLC cell lines; **b** SNHG6 overexpression inhibited cell apoptosis of A549 and SPCA1 cells; **c** SNHG6 knockdown decreased NSCLC cells in the S phase and increased the cells in the G0/G1 phase; **d** The overexpression of SNHG6 promoted G1/S transition; **e** SNHG6 suppression downregulated the expression of cyclin D1, CDK2, CDK4, cyclin A2, and cyclin E1 and SNHG6 overexpression represented the opposite effects. The results were from three independent experiments. Data were analyzed by unpaired student’s *t*-test. ***P < 0.001, **P < 0.01, *P < 0.05. Variables were presented as the mean ± SD.

To further investigate the function of SNHG6, we carried out flow cytometry assays. The cell apoptosis assays revealed that SNHG6 knockdown increased the apoptosis rate of A549 and SPCA1 cells (Fig. 3a), and SNHG6 overexpression significantly decreased the apoptotic cells (Fig. 3b). The depletion of SNHG6 remarkably decreased the proportion of cells in the S phase and induced G0/G1 arrest in both cell lines (Fig. 3c). On the contrary, SNHG6 overexpression facilitated the G1/S transition (Fig. 3d). Subsequently, we detected the predominant cyclin-dependent kinases (CDKs) and cyclins (CCNs) in the G0/G1 phase to further validate the regulatory role of SNHG6 in the cell cycle [19]. Western blot assays showed that SNHG6 silencing suppressed the expression of cyclin D1, CDK4, CDK2, cyclin E1, and cyclin A2, while SNHG6 overexpression manifested the inverse results (Fig. 3e). Taken together, the flow cytometry assays validated the tumorigenic implication of SNHG6 in cell cycle and cell apoptosis.

### SNHG6 epigenetically suppressed p27 expression by recruiting EZH2 to the promoter region of p27

**Fig. 4:**
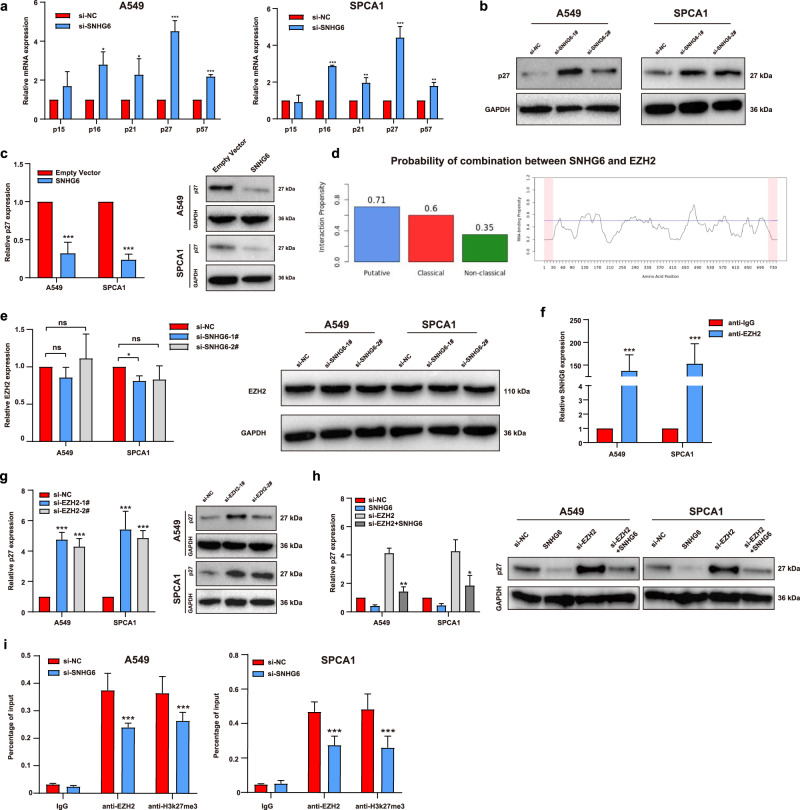
SNHG6 epigenetically silenced p27 expression by recruiting EZH2 to its promoter region. **a** The relative mRNA expression of cyclin-dependent kinase inhibitors after SNHG6 silencing; **b** p27 was upregulated after SNHG6 knockdown in protein level; **c** Overexpression of SNHG6 downregulated the p27 expression in mRNA and protein levels; **d** SNHG6 had a positive tendency to interact with EZH2 according to catRAPID and the potential binding sites were illustrated; **e** Knockdown of SNHG6 exerted no influence on the expression of EZH2 in mRNA and protein levels; **f** RIP assays were employed to verify the interaction between SNHG6 and EZH2 in A549 and SPCA1; **g** Knockdown of EZH2 upregulated the mRNA and protein expressions of p27 in both cell lines; **h** SNHG6 upregulation rescued the increased expression of p27 arisen from EZH2 inhibition. **i** ChIP assays showed that the knockdown of SNHG6 decreased the enrichment of EZH2 and H3K27me3 at the promoter region of p27. The results were from three independent experiments. Data were analyzed by unpaired student’s *t*-test. ***P < 0.001, **P < 0.01, *P < 0.05. Variables were presented as the mean ± SD.

The cell cycle assays demonstrated that SNHG6 could influence the process of G1/S transition in NSCLC cells (Fig. 3c, d). As previous investigations reported, G1/S transition was largely regulated by cyclin-dependent kinase inhibitors (CKIs) [20–24]. Thus, we detected the expression of CDKN2B (p15), CDKN2A (p16), CDKN1A (p21), CDKN1B (p27), and CDKN1C (p57) after SNHG6 knockdown (Fig. 4a). It indicated that p27 was the noblest upregulated gene after SNHG6 silencing in both NSCLC cells. Similarly, we further found that SNHG6 knockdown upregulated p27 expression at the protein level (Fig. 4b). On the contrary, SNHG6 overexpression downregulated p27 mRNA and protein expression (Fig. 4c). Consequently, we speculated that SNHG6 might function through regulating p27 expression in NSCLC progression.

It reported that lncRNAs could function in cooperation with chromatin-modifying enzymes to promote epigenetic activation or silencing of the target genes [25], such as Polycomb repressive complex (PRC) [26], DNA methyltransferase 1 (DNMT1) [27], and lysine-specific demethylase 1 (LSD1) [28]. Enhancer of Zeste Homolog 2 (EZH2) is the catalytic subunit of PRC2 that regulates downstream genes by trimethylation of Lys-27 in histone 3 (H3K27me3) [29]. Additional studies manifested that EZH2 could serve as an RNA-binding protein (RBP) in tumor progression [30–32]. We therefore examined whether SNHG6 could bind with EZH2 using the RNA-protein interaction prediction RPIseq (http://pridb.gdcb.iastate.edu/RPISeq/). The results showed that the prediction using RF classifier was 0.7 and the SVM classifier was 0.96 (The predictions with probabilities >0.5 were considered positive). Using another predictor catRAPID (http://s.tartaglialab.com/page/catrapid_omics2_group), we observed that the overall interaction score between SNHG6 and EZH2 was 0.54 (Fig. 4d, putative = 0.71, classical = 0.6, and non-classical = 0.3, the interaction score >0.5 suggested RNA-binding potential). The potential binding loci were illustrated in Fig. 4d as well. Subsequently, we assessed the EZH2 level after SNHG6 knockdown by qPCR and western blot assays. It indicated that EZH2 showed no significant variations at mRNA and protein levels after SNHG6 silencing (Fig. 4e). We further carried out the RIP assays to validate the interaction between SNHG6 and EZH2. It implied that the enrichment of SNHG6 was prominently higher in the anti-EZH2 group compared with anti-IgG in A549 and SPCA1 cell lines (Fig. 4f). All the findings above manifested that SNHG6 could directly interact with EZH2.

Furthermore, two siRNAs were synthesized to downregulate EZH2 in A549 and SPCA1 (Fig. S1c). Consistent with the silencing of SNHG6, EZH2 knockdown visibly upregulated the mRNA and protein expressions of p27 (Fig. 4g). Moreover, EZH2 inhibition partially rescued the decreasing p27 expression mediated by SNHG6 overexpression at mRNA and protein levels (Fig. 4h). In addition, ChIP assays showed that downregulation of SNHG6 inhibited recruiting EZH2 to p27 promoter and suppressed H3K27me3 modification (Fig. 4i). The above findings suggested that SNHG6 epigenetically regulated p27 by interacting with EZH2 in NSCLC.

### SNHG6 controlled the cell cycle by regulating p27 and lower expression of p27 was associated with a poor prognosis in NSCLC

**Fig. 5:**
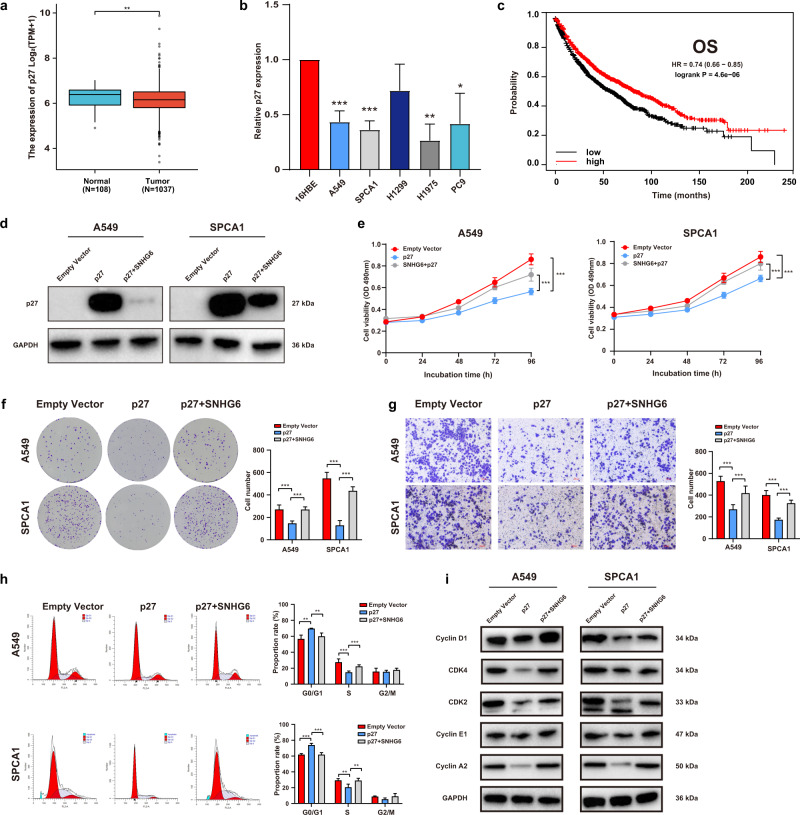
p27 overexpression suppressed cell biological function, which was partially rescued by SNHG6 overexpression. **a**, **b** p27 was downregulated in NSCLC tissues and cells (A549, SPCA1, and, H1975); **c** lower expression of p27 indicated a poorer prognosis (log-rank test); **d** Transfection with pcDNA3.1-p27 in NSCLC cells efficiently upregulated the p27 protein expressions while overexpression of SNHG6 reversed the upregulation; **e**–**g** Overexpression of p27 inhibited cell proliferation (**e**: MTT; **f**: colony formation) and migration (**g**) while SNHG6 recovered the suppressed cell function. **h** SNHG6 partly retrieved the declined cell number in the S phase and the increased cell number in the G0/G1 phase that was mediated by p27 overexpression; **i** p27 downregulated the expression of CCNs and CDKs that function in G1/S transition and SNHG6 partially retrieved the decline. The results were from three independent experiments. Data were analyzed by unpaired student’s *t*-test. ***P < 0.001, **P < 0.01, *P < 0.05. Variables were presented as the mean ± SD.

We evaluated the differential expression level of p27 in NSCLC using the TCGA database. It showed that p27 was aberrantly downregulated in NSCLC tumor tissues compared with normal tissues (Fig. 5a, Normal = 108 vs Tumor = 1037, *P* < 0.01). Likewise, p27 was downregulated in NSCLC cell lines (A549, SPCA1, H1975, and PC9) (Fig. 5b). Additionally, the low expression of p27 was associated with a dismal NSCLC prognosis (Fig. 5c, Overall survival, *P* < 0.001, HR = 0.74 (0.66–0.85)). The results above highlighted that p27 served as a tumor suppressor in NSCLC progression.

To explore the biological function of p27 in NSCLC, we constructed the overexpression plasmid pcDNA3.1-p27. As Fig. S1d manifested, pcDNA3.1-p27 efficiently upregulated p27 expression in A549 and SPCA1 cells. Subsequently, we performed several rescue assays. The results of western blot assays showed that co-transfection of pcDNA3.1-SNHG6 and pcDNA3.1-p27 partially reversed the increased expression of p27 mediated by pcDNA3.1-p27 transfection alone (Fig. 5d). MTT assays revealed that p27 overexpression suppressed cell proliferation of A549 and SPCA1 while SNHG6 partially rescued the decreased cell viability mediated by p27 (Fig. 5e). A similar result was observed in colony-forming assays (Fig. 5f). Furthermore, p27 upregulation weakened cell migration while SNHG6 upregulation retrieved such decline (Fig. 5g). The cell cycle assays demonstrated that p27 upregulation increased the proportion of cells in the G0/G1 stage and decreased cell proportion in the S phase. SNHG6 overexpression partially reversed the effects arising from p27 (Fig. 5h). In addition, p27 downregulated the expressions of Cyclin D1, CDK4, CDK2, Cyclin E1, and Cyclin A2, and SNHG6 could partly retrieve such decline in A549 and SPCA1 cells (Fig. 5i). In summary, p27 restrained the cellular function of NSCLC cells, and SNHG6 rescued such inhibition mediated by p27.

### SNHG6 knockdown inhibited NSCLC tumor growth in vivo

**Fig. 6:**
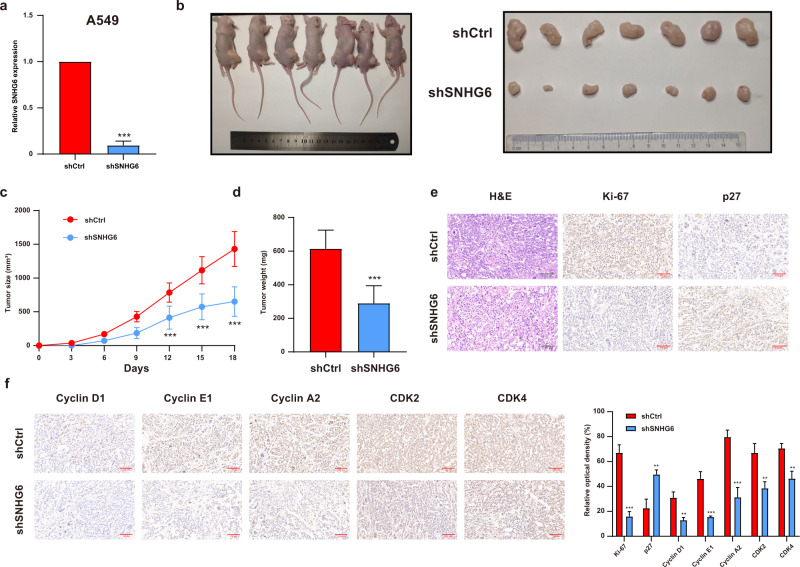
SNHG6 knockdown restrained tumor growth in vivo. **a** shSNHG6 downregulated the expression of SNHG6 in A549 cells; **b** The stably transfected A549 cells were subcutaneously injected into the back of nude mice, right for the shCtrl group and left for the shSNHG6 severally; **c** The volumes of tumors were measured every 3 days and the growth curves of the shCtrl group and shSNHG6 group were exhibited; **d** The weights of dissected tumors were detected; **e** The tumor sections were undergone H&E, Ki-67, and p27 staining; **f** The IHC staining of cyclin D1, cyclin E1, cyclin A2, CDK2, and CDK4 were conducted. Data were analyzed by unpaired student’s *t*-test. ***P < 0.001, **P < 0.01. Variables were presented as the mean ± SD.

Oncogenesis assay in nude mice was conducted to observe the cell proliferation abilities in vivo. As Fig. 6a illustrated, stably transfected shSNHG6 drastically decreased the expression of SNHG6 in A549 cells. The tumors of the shSNHG6 group had a slower speed of growth in comparison with the shCtrl group, which was reflected by the tumor volume (Fig. 6b, c). The averaged tumor weight measured on the 18th day was significantly lower in the shSNHG6 group compared with the shCtrl group (Fig. 6d). Furthermore, the positive rate of ki-67 was markedly decreased in tumors formed from A549 cells that were stably transfected with shSNHG6 compared with those from shCtrl. The tumors in the shSNHG6 group represented a higher positive rate of p27 than the control (Fig. 6e). In addition, we performed the immunohistochemistry (IHC) staining of Cyclin D1, Cyclin E1, Cyclin A2, CDK2, and CDK4. As Fig. 6f demonstrated, the positive rate of Cyclin D1, Cyclin E1, Cyclin A2, CDK2, and CDK4 were lowered after SNHG6 knockdown. These findings collectively indicated that SNHG6 reduction inhibited tumor development of NSCLC by affecting cell cycle progression in vivo.

## Discussion

Previous investigations have verified that lncRNAs played crucial roles in NSCLC progression [33]. It indicated that lncRNA regulated NSCLC tumor biological function, including proliferation, migration, invasion, and apoptosis in a significant and flexible manner. For example, linc00673 regulated epithelial-mesenchymal transition (EMT) through sponging miR-150-5p in NSCLC [34]. JPX regulated tumorigenesis and metastasis via JPX/miR-33a-5p/Twist1 axis and activated Wnt/β-catenin signaling in lung cancer [35]. LY6K-AS interacted with 14-3-3 proteins to regulate the transcription of kinetochore and mitotic checkpoint proteins in lung adenocarcinoma [36].

In this study, we selected SNHG6 as the research object by screening the differential-expressed lncRNAs in GEO (GSE33532 and GSE29249). SNHG6, located in 8q13.1, has been validated to play a carcinogenic role in various malignancies and could serve as a predictive factor related to the prognosis [11–14]. Consistent with previous studies, we found SNHG6 was markedly upregulated in NSCLC tissues and cell lines. The upregulation of SNHG6 was correlated with a poorer prognosis of NSCLC patients. Additionally, we are the first to find that copy number amplification is an important cause of SNHG6 activation in NSCLC. In fact, many oncogenic lncRNAs were upregulated in malignancies, which was resulted from gene copy number variation (CNV) as well [37]. Zhong WW et al. comprehensively analyzed numerous lncRNAs with CNV in bladder cancer [38]. Our previous study also found that the amplification of SNHG17 could lead to the upregulation of SNHG17 in NSCLC [39]. Subsequently, we conducted a series of functional assays. The results revealed that the knockdown of SNHG6 suppressed the proliferation and migration abilities of NSCLC cells, inhibited G1/S transition, and promoted cell apoptosis. Upregulation of SNHG6 exhibited the contrary tendency in NSCLC. The experiments on nude mice also suggested that SNHG6 regulated tumor growth in vivo.

CKIs are a class of genes that encode proteins that bind to and inactivate CCN/CDK complexes, which serve as suppressors in tumor progression [40, 41]. According to the forepassed studies, CKIs could regulate the G1/S transition in the cell cycle [42–44]. In this study, the G1/S transition was inhibited after SNHG6 knockdown. Hence, we detected the relative expression of CKIs (p15, p16, p21, p27, and p57) after SNHG6 downregulation and the result revealed that p27 was distinctly upregulated in both NSCLC cell lines. Reasonably, we hypothesized SNHG6 regulated p27 expression, thereby regulating the cell cycle, to promote the progression of NSCLC.

EZH2 is the catalytic subunit of Polycomb Repressive Complex 2 (PRC2) and functions as a methyltransferase to mediate H3K27 trimethylation in the promoter region of target genes [45, 46]. It reported that several lncRNAs could interact with EZH2 to epigenetically suppress the expression of the downstream gene [47–49]. For instance, CASC9 promoted esophageal squamous cell carcinoma (ESCC) progression by negatively regulating PDCD4 expression through recruiting EZH2 [50]. In this study, we found SNHG6 could directly bind to EZH2, and the knockdown of EZH2 upregulated the expression of p27, which was consistent with SNHG6 knockdown. Additionally, co-transfection of SNHG6 and p27 was able to partially reverse the overexpression mediated by p27 transfection alone. Subsequently, we performed ChIP assays and discovered that SNHG6 recruited EZH2 to the p27 promoter region and increased the H3K27me3 enrichment of its promoter. Finally, we conducted several rescue assays to validate the regulatory function of SNHG6 and p27 in NSCLC. Briefly, SNHG6 rescued the p27 expression and impaired cellular function mediated by p27 overexpression.

Additionally, there existed alterations of CDK/cyclin complexes expression in the cells of G0/G1 arrest [51, 52]. In the present study, the expression of Cyclin D1, Cyclin A2, Cyclin E1, CDK2, and CDK4 were downregulated after SNHG6 knockdown in vivo and in vitro. p27 overexpression downregulated the CCN/CDK expression and SNHG6 overexpression partially retrieved such downregulation. These findings suggested that SNHG6 can regulate the G1/S transition of the cell cycle of NSCLC by targeting the expression of p27.

Taken together, this study convinced us that gene amplification-driven SNHG6 participated in the cell cycle through SNHG6/EZH2/p27 axis to promote NSCLC progression. Our study may disclose a new therapeutic target in NSCLC treatment.

## Methods and materials

### Cell culture and transfections

Human NSCLC cell lines (A549, SPCA1, H1299, H1975, and PC9) and human bronchial epithelial cell line (16HBE) were purchased from the cell bank of the Chinese Science Academy. SPCA1, PC9, and 16HBE were cultured in Dulbecco’s modified Eagle’s medium (DMEM) (Gibco, Rockville, USA) supplemented with 10% fetal bovine serum (FBS) (Biological Industries, Israel) and 1% Penicillin-Streptomycin (Gibco, Rockville, USA). A549, H1299, and H1975 were incubated in Roswell Park Memorial Institute (RPMI) 1640 medium (Gibco, Rockville, USA) that contained the same ingredients. All the cell lines were incubated in a man-made environment with 5% CO_2_ and 37 °C and excluded from mycoplasma infection. The sequences of small interfering RNAs (siRNAs) for SNHG6, EZH2, and short hairpin RNA (shRNA) for SNHG6 were listed in Tables S1, S2. The cDNA encoding SNHG6 and p27 were amplified and cloned into pcDNA3.1 vector (Invitrogen, Carlsbad, USA) to form the pcDNA3.1-SNHG6 and pcDNA3.1-p27 overexpression plasmid. SiRNA, shRNA, and plasmid transfections were performed using Lipofectamine 3000 (Invitrogen, Carlsbad, USA) in opti-MEM medium (Gibco, Rockville, USA). The transfection by the lentivirus vector was detailed in the part of the animal experiment.

### RNA extraction and quantitative real‐time PCR (qRT-PCR)

The total RNAs were extracted from cells in the logarithmic growth phase using TRIzol reagent (Invitrogen, Carlsbad, USA) after 48 h transfection. PrimeScriptTM II Reverse Transcriptase (Takara, Tokyo, Japan) was used to reversely transcribe the extracted RNAs to corresponding cDNAs. qRT-PCR assays were performed using SYBR Green PCR Kit (Takara, Tokyo, Japan) on QuantStudio 7 (Thermo Fisher, California, USA) and the analyses of relative gene expression were performed using the 2^-ΔΔCT^ method. The primers are listed in Table S3. All the primers were synthesized by GENERAY, Shanghai, China.

### MTT assay

The transfected cells were seeded in 96-well plates at the density of 2 × 10^3^ per well. At the time of 0, 24, 48, 72, and 96 h, the cells will be incubated in 200 μL fresh complete medium that contains 20 μL MTT (0.5 mg/mL) for 3.5 h. Then, the medium was discarded and 150 μL DMSO per well was added to dissolve the formazan that formed from MTT. The cell viability was measured at the absorbance of 490 nm.

### Colony-forming assay

The transfected cells were planted in 6-well plates at the density of 2 × 10^3^ per well and incubated in a complete medium for 12 days. The medium was replaced every 4 days. On the 12th day, the medium was removed and colonies were fixed with 4% paraformaldehyde at room temperature for 20 min. Following, the colonies were stained with 0.5% crystal violet (Beyotime, Shanghai, China). The number of colonies was counted by ImageJ software.

### 5‑Ethynyl‑2′‑deoxyuridine (EdU) assay

The transfected cells were planted in 96-well plates at the density of 1 × 10^5^ per well and incubated in a complete medium for 2 days. The assays were carried out using YF®555 Click-iT EdU Imaging Kits (US EVERBRIGHT, China) according to the instructions of the manufacturer. Then, the graphs of stained cells were captured under the inverted fluorescence microscope. The proliferation ability was assessed using the formula: Proliferation rate = the number of EdU stained cells/the number of DAPI stained cells.

### Transwell assays

In total, 5 × 10^4^ NSCLC cells in 300 μl serum-free medium were seeded in the upper compartment of the Transwell apical chamber (Corning Life Sciences, Corning, NY, USA), and 700 μl complete medium was placed in the lower chamber. After 24–48 h, the cells were fixed with 4% paraformaldehyde for 20 min, and those migrated to the bottom surface of the chamber were stained with crystal violet (Beyotime, Shanghai, China). Five microscopic fields per chamber were observed for counting the migratory cells (at ×100 magnification). ImageJ software was used for counting.

### Flow cytometry assays

For cell cycle assays, the adherent cells were collected 48 h after transfection and fixed in 80% ethanol. Then, the cells were stained with Propidium Iodide (PI). For cell apoptosis assays, the suspending cells and adherent cells were both collected. The assays were performed using YF®488-Annexin V and PI Apoptosis Kit (US EVERBRIGHT, Suzhou, China) under the instructions of the manufacturer. The detection was carried out by FACSCalibur (BD, New Jersey, USA). Data analysis of cell cycle distribution and apoptosis rate were performed using FlowJo software version 10.8.0 (BD, New Jersey, USA).

### Western blot assays

RIPA lysis buffer (Beyotime, Shanghai, China) containing Protease Inhibitor Cocktail (MCE, Shanghai, China) (volume ratio = 100:1) was used to extract proteins which were later stored at −20 °C. Proteins were separated by 4–20% SurePAGE (GeneSript, Nanjing, China) and transferred to PVDF membranes (Millipore, Massachusetts, USA) after electrophoresis. The membranes were then blocked in TBST with 5% skim milk powder for 2 h. After blocking, the membranes were incubated in antibodies purchased from Abcolonal (Wuhan, China) as follows: p27 (1:800 dilution), CDK2 (1:1000 dilution), CDK4 (1:1000 dilution), CCND1 (1:1000 dilution), CCNE1 (1:1000 dilution), CCNA2 (1:1000 dilution), GAPDH (1:2000 dilution). After overnight incubation, the membranes were incubated in HRP Goat Anti-Rabbit IgG (H + L) (1:10000 dilution) for 2 h. Finally, the bands were detected under GelDoc XR + ((Bio-Rad, California, USA) through Clarity ECL Substrate (Bio-Rad, California, USA).

### RNA Immunoprecipitation (RIP)

In brief, the control of IgG antibody and EZH2 antibody were combined with the magnetic beads and incubated with cell lysates of A549 or SPCA1 at 4 °C overnight. Then, the protein was digested by Protein K and the total RNA was extracted by phenol-chloroform isoamylol (25:24:1). Total RNAs were used as input. Reverse transcription and qRT-PCR were further performed to detect SNHG6 expression.

### Chromatin immunoprecipitation (ChIP)

The ChIP assays were performed by EZ-Magna ChIP^TM^ A/G chromatin immunoprecipitation kit according to the manufacturer’s protocols. The anti-EZH2 and anti-H3K27me3 were purchased from Abclonal. The purified immunoprecipitated DNAs from the si-NC and si-SNHG6 groups were detected by qRT-PCR. The primer for ChIP was shown in Table S3.

### Animal experiment

We constructed the stably transfected A549 cell lines with shSNHG6 and negative control (shCtrl) for in vivo experiments. The lentivirus-coated shRNAs containing the green fluorescent protein (GFP) were synthesized by GENECHEM (Shanghai, China) and the transfection was performed according to the instructions of the manufacturer. The transfection efficiency was determined by observing the fluorescence intensity under the microscope and the knockdown efficiency of shSNHG6 was further accurately evaluated by qRT-PCR.

Seven BALB/c male nude mice (6 weeks old) were purchased from Weitonglihua (Beijing, China). About 1 × 10^6^ stably transfected A549 cells were inoculated subcutaneously into the back of nude mice, right for the shSNHG6 group and left for the shCtrl group, respectively. The tumor volume was monitored every 3 days and calculated based on the following formula: V = larger diameter × (smaller diameter)^2^/2 every 3 days. On the 15th day, all the mice were euthanized and the tumors were taken out for weighing. The tumor tissues were fixed with 4% paraformaldehyde for hematoxylin-eosin (HE) and Ki-67 staining. This study was performed according to the Guide for the Care and Use of Laboratory Animals of the National Institutes of Health. The study protocol was approved by the Committee on the Ethics of Animal Experiments of Nanjing Medical University.

### Statistical analysis

Differential expression analysis and statistical significance of differences between the different groups were performed using unpaired student’s *t*-test and the data passed normal distribution detection. Kaplan–Meier method and log-rank test were adopted to evaluate the associations between SNHG6/p27 expression and NSCLC prognosis. All the analyses were performed based on R 3.6.3 and Graphpad Prism 8.0. *P* < 0.05 was considered significant (*, **, *** represent *P* < 0.05, *P* < 0.01, and *P* < 0.001 respectively). All the data were from three independent experiments.

## Supplementary information

The confirming email for author list change

Original Data File

SUPPLEMENTAL MATERIAL

## Data Availability

The data underlying this article will be shared on reasonable request to the corresponding author.
