# Supplementary material for: Gene amplification-driven lncRNA SNHG6 promotes tumorigenesis via epigenetically suppressing p27 expression and regulating cell cycle in non–small cell lung cancer
Source: Cell Death Discov. 2022 Dec 9;8:485. doi: 10.1038/s41420-022-01276-y (PMC9734177; doi:10.1038/s41420-022-01276-y)
Supplement: Supplementary file 1 — The confirming email for author list change [file 41420_2022_1276_MOESM1_ESM.pdf]

回复：**Author list change for agreement** ☆

发件人：王琦 <njmuwq@foxmail.com>
时　间：2022年11月25日（星期五）下午12：56
收件人：吴卫兵 <wuweibing95@126.com>; 张二宝 <erbaozhang@njmu.edu.cn>; 张伟 <jasonfreerice@foxmail.com>; tanghuangmail <tanghuangmail@163.com>; jsnydandan <jsnydandan@sina.com> [再发一封给所有人]

纯文本 |

发送状态：投递成功 [查看详情]　　什么是发送状态?

OK，I agree to the new order of author list.

王琦
njmuwq@foxmail.com

---------------- 原始邮件 ----------------

发件人: "吴卫兵" <wuweibing95@126.com>;
发送时间: 2022年11月25日(星期五) 中午11:13
收件人: "erbaozhang@njmu.edu.cn"<erbaozhang@njmu.edu.cn>;"jasonfreerice@foxmail.com"<jasonfreerice@foxmail.com>;"tanghuangmail@163.com"<tanghuangmail@163.com>;"jsnydandan@sina.com"<jsnydandan@sina.com>;"王琦"<njmuwq@foxmail.com>;
主题: Author list change for agreement

Dear co-authors:

Our submission to ***Cell Death Discovery*** entitled "Gene amplification-driven lncRNA SNHG6 promotes tumorigenesis via epigenetically suppressing p27 expression and regulating cell cycle in non-small cell lung cancer" **(ID: *CDDISCOVERY-22-3911R1*)** has been provisionally accepted by the editorial office. In the process of revision, Dr. Dandan Yin made lot contributions to this work. After all of our authors had discussed it, we agreed to add Dandan Yin as co-first author. The current author order is "Qi Wang#, Wei Zhang#, Dandan Yin#, Zaibin Tang, Erbao Zhang*, Weibing Wu*".

Now we need to reconfirm by email. Please reply to the email with your confirmation. Thanks!

Yours sincerely,

Weibing Wu

wuweibing95
wuweibing95@126.com

Re:Author list change for agreement ☆

发件人：张二宝 <erbaozhang@njmu.edu.cn>
时　间：2022年11月25日（星期五）上午11：20
收件人：吴卫兵 <wuweibing95@126.com>
抄　送：张伟 <jasonfreerice@foxmail.com>; tanghuangmail <tanghuangmail@163.com>; jsnydandan <jsnydandan@sina.com>; 王琦 <njmuwq@foxmail.com>

纯文本 |

Ok, I agree with this order of authorship.

Erbao Zhang, Ph.D.
Department of Epidemiology, Center for Global Health, School of Public Health, Nanjing Medical University;
Jiangsu Key Lab of Cancer Biomarkers, Prevention and Treatment, Collaborative Innovation Center for Cancer Personalized Medicine,
Nanjing Medical University.

发件人："吴卫兵" <wuweibing95@126.com>
发送日期：2022-11-25 11:13:30
收件人："erbaozhang@njmu.edu.cn" <erbaozhang@njmu.edu.cn>,"jasonfreerice@foxmail.com" <jasonfreerice@foxmail.com>,"tanghuangmail@163.com" <tanghuangmail@163.com>,"jsnydandan@sina.com" <jsnydandan@sina.com>,"njmuwq@foxmail.com" <njmuwq@foxmail.com>
主题：Author list change for agreement

Dear co-authors:

Our submission to ***Cell Death Discovery*** entitled "Gene amplification-driven lncRNA SNHG6 promotes tumorigenesis via epigenetically suppressing p27 expression and regulating cell cycle in non–small cell lung cancer" **(ID: *CDDISCOVERY-22-3911R1*)** has been provisionally accepted by the editorial office. In the process of revision, Dr. Dandan Yin made lot contributions to this work. After all of our authors had discussed it, we agreed to add Dandan Yin as co-first author. The current author order is "Qi Wang[#], Wei Zhang[#], Dandan Yin[#], Zaibin Tang, Erbao Zhang[*], Weibing Wu[*]".

Now we need to reconfirm by email. Please reply to the email with your confirmation. Thanks!

Yours sincerely,

Weibing Wu

wuweibing95
wuweibing95@126.com

回复：Author list change for agreement ☆

发件人：**jsnydandan** <jsnydandan@sina.com>

时　间：2022年11月25日（星期五）上午11：58

收件人：吴卫兵 <wuweibing95@126.com>; 张二宝 <erbaozhang@njmu.edu.cn>; 张伟 <jasonfreerice@foxmail.com>; tanghuangmail <tanghuangmail@163.com>; 王琦 <njmuwq@foxmail.com>

纯文本 |

That is OK. I agree with this author order.

Dandan Yin

----- 原始邮件 -----

发件人：吴卫兵 <wuweibing95@126.com>

收件人："erbaozhang@njmu.edu.cn" <erbaozhang@njmu.edu.cn>, "jasonfreerice@foxmail.com" <jasonfreerice@foxmail.com>, "tanghuangmail@163.com" <tanghuangmail@163.com>, "jsnydandan@sina.com" <jsnydandan@sina.com>, "njmuwq@foxmail.com" <njmuwq@foxmail.com>

主题：Author list change for agreement

日期：2022年11月25日 11点13分

Dear co-authors:

Our submission to ***Cell Death Discovery*** entitled "Gene amplification-driven lncRNA SNHG6 promotes tumorigenesis via epigenetically suppressing p27 expression and regulating cell cycle in non–small cell lung cancer" **(ID: *CDDISCOVERY-22-3911R1*)** has been provisionally accepted by the editorial office. In the process of revision, Dr. Dandan Yin made lot contributions to this work. After all of our authors had discussed it, we agreed to add Dandan Yin as co-first author. The current author order is "Qi Wang[#], Wei Zhang[#], Dandan Yin[#], Zaibin Tang, Erbao Zhang[*], Weibing Wu[*]".

Now we need to reconfirm by email. Please reply to the email with your confirmation. Thanks!

Yours sincerely,

Weibing Wu

wuweibing95

wuweibing95@126.com

回复: Author list change for agreement ☆

发件人: **tanghuangmail** <tanghuangmail@163.com>

时　间: 2022年11月25日 (星期五) 下午12：12

收件人: 吴卫兵 <wuweibing95@126.com>; 张二宝 <erbaozhang@njmu.edu.cn>; 张伟 <jasonfreerice@foxmail.com>; jsnydandan <jsnydandan@sina.com>; 王琦 <njmuwq@foxmail.com>

纯文本 |

Thanks for your email, I agree with this order of authorship.

-------- 原始邮件 --------

发件人: 吴卫兵 <wuweibing95@126.com>

日期: 2022年11月25日周五 11:13

收件人: erbaozhang@njmu.edu.cn, jasonfreerice@foxmail.com, tanghuangmail@163.com, jsnydandan@sina.com, njmuwq@foxmail.com

主 题: Author list change for agreement

Dear co-authors:

Our submission to ***Cell Death Discovery*** entitled "Gene amplification-driven lncRNA SNHG6 promotes tumorigenesis via epigenetically suppressing p27 expression and regulating cell cycle in non-small cell lung cancer" **(ID: *CDDISCOVERY-22-3911R1*)** has been provisionally accepted by the editorial office. In the process of revision, Dr. Dandan Yin made lot contributions to this work. After all of our authors had discussed it, we agreed to add Dandan Yin as co-first author. The current author order is "Qi Wang[#], Wei Zhang[#], Dandan Yin[#], Zaibin Tang, Erbao Zhang[*], Weibing Wu[*]".

Now we need to reconfirm by email. Please reply to the email with your confirmation. Thanks!

Yours sincerely,

Weibing Wu

wuweibing95

wuweibing95@126.com

签名由 网易邮箱大师 定制

回复：Author list change for agreement ☆

发件人：张伟 <jasonfreerice@foxmail.com>

时　间：2022年11月25日（星期五）下午12：33

收件人：吴卫兵 <wuweibing95@126.com>; 张二宝 <erbaozhang@njmu.edu.cn>; tanghuangmail <tanghuangmail@163.com>; jsnydandan <jsnydandan@sina.com>; 王琦 <njmuwq@foxmail.com>

纯文本 |

I confirm all co-authors of the article and agree with the order

胸外科张伟

jasonfreerice@foxmail.com

江苏省人民医院/南京医科大学

研究生

------------------ 原始邮件 ------------------

发件人: "吴卫兵" <wuweibing95@126.com>;

发送时间: 2022年11月25日(星期五) 中午11:13

收件人: "erbaozhang@njmu.edu.cn"<erbaozhang@njmu.edu.cn>;"胸外科张伟"<jasonfreerice@foxmail.com>;"tanghuangmail@163.com"<tanghuangmail@163.com>;"jsnydandan@sina.com"<jsnydandan@sina.com>;"njmuwq@foxmail.com"<njmuwq@foxmail.com>;

主题: Author list change for agreement

Dear co-authors:

Our submission to ***Cell Death Discovery*** entitled "Gene amplification-driven lncRNA SNHG6 promotes tumorigenesis via epigenetically suppressing p27 expression and regulating cell cycle in non-small cell lung cancer" **(ID: *CDDISCOVERY-22-3911R1*)** has been provisionally accepted by the editorial office. In the process of revision, Dr. Dandan Yin made lot contributions to this work. After all of our authors had discussed it, we agreed to add Dandan Yin as co-first author. The current author order is "Qi Wang#, Wei Zhang#, Dandan Yin#, Zaibin Tang, Erbao Zhang*, Weibing Wu*".

Now we need to reconfirm by email. Please reply to the email with your confirmation. Thanks!

Yours sincerely,

Weibing Wu

wuweibing95

wuweibing95@126.com
