# Supplementary figures and images for: Gene amplification-driven lncRNA SNHG6 promotes tumorigenesis via epigenetically suppressing p27 expression and regulating cell cycle in non–small cell lung cancer
Source: Cell Death Discov. 2022 Dec 9;8:485. doi: 10.1038/s41420-022-01276-y (PMC9734177; doi:10.1038/s41420-022-01276-y)

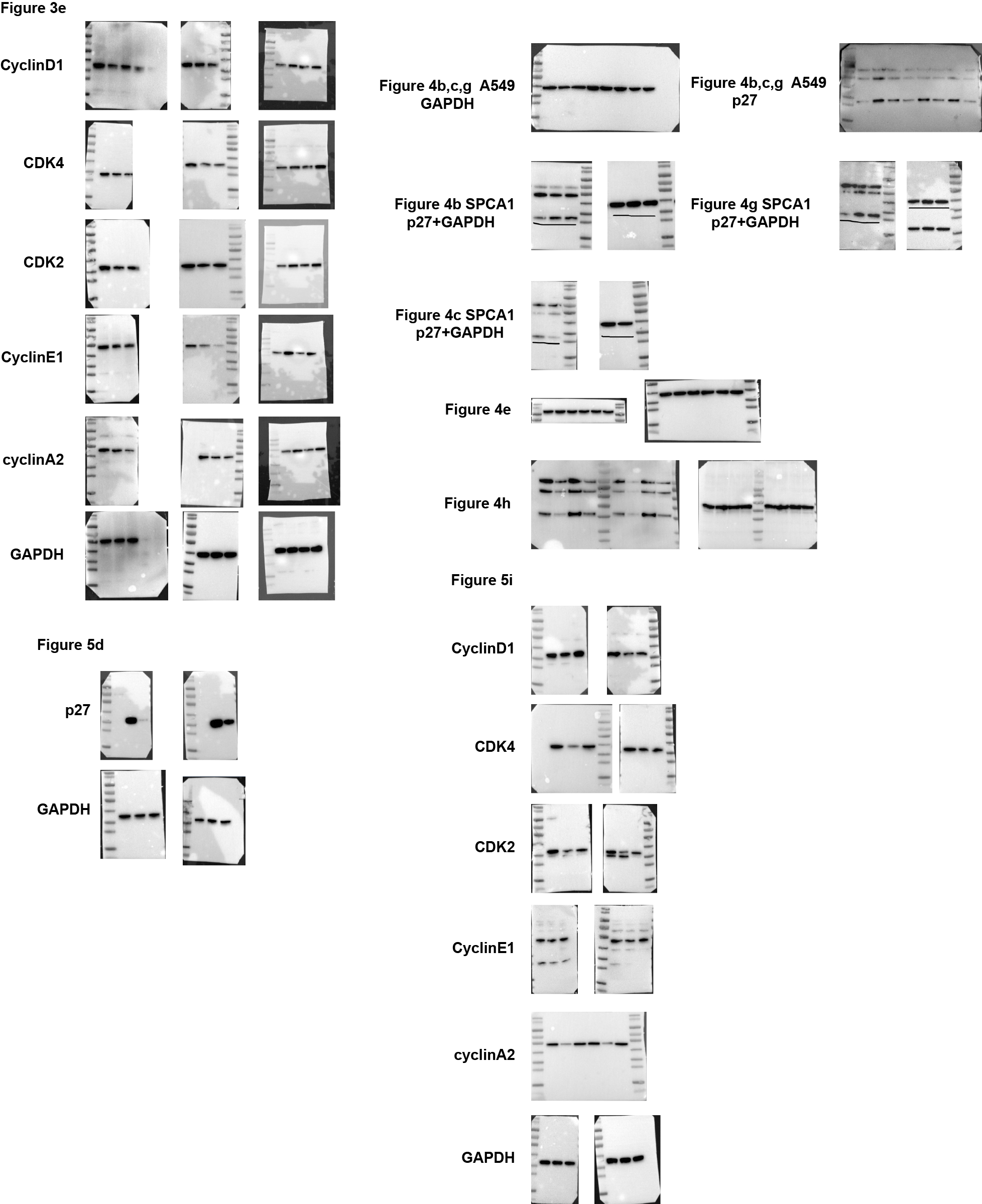

Supplement: Supplementary file 2 — Original Data File [file 41420_2022_1276_MOESM2_ESM.tif]
