# Supplementary material for: Gene amplification-driven lncRNA SNHG6 promotes tumorigenesis via epigenetically suppressing p27 expression and regulating cell cycle in non–small cell lung cancer
Source: Cell Death Discov. 2022 Dec 9;8:485. doi: 10.1038/s41420-022-01276-y (PMC9734177; doi:10.1038/s41420-022-01276-y)
Supplement: Supplementary file 3 — SUPPLEMENTAL MATERIAL [file 41420_2022_1276_MOESM3_ESM.docx]

**Supporting information**

**Figure S1. The transfection efficiency of siRNAs and overexpression plasmids.**

**Table S1: The sequences of siRNAs**

**Table S2: The sequence of shSNHG6**

**Table S3: The sequences of primers**

**
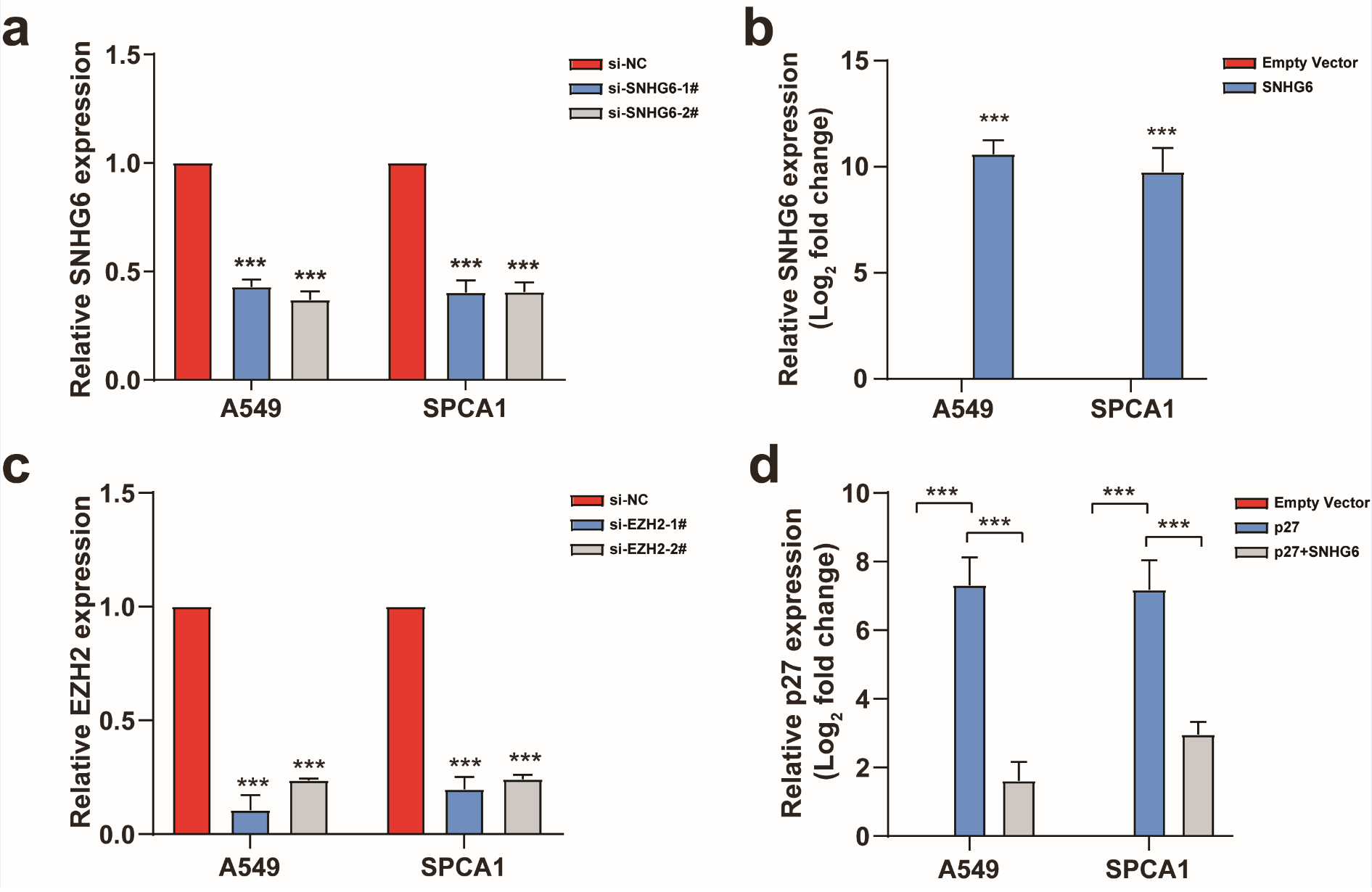
**

**a.** si-SNHG6 efficiently downregulated SNHG6 expression in A549 and SPCA1; **b.** pcDNA3.1-SNHG6 markedly upregulated SNHG6 expression; **c.** si-EZH2 significantly downregulated EZH2 expression; **d.** pcDNA3.1-p27 upregulated the expression of p27 which was partially reversed by SNHG6 overexpression.

**Table S1: The sequences of siRNAs**

|  | Sequences (5’-3’) |
| --- | --- |
| si-SNHG6-1# | sense: AGAAAGCCUUUGAGGUGAAGGUGUATT |
|  | antisense: UACACCUUCACCUCAAAGGCUUUCUTT |
| si-SNHG6-2# | sense: CGGCAUGUAUUGAGCAUAUAGGUUGTT |
|  | antisense: CAACCUAUAUGCUCAAUACAUGCCGTT |
| si-EZH2 -1# | sense: GGAUACAGCCUGUGCACAUTT |
|  | antisense: AUGUGCACAGGCUGUAUCCTT |
| si-EZH2 -2# | sense: GCUCUAGACAACAAACCUUTT |
|  | antisense: AAGGUUUGUUGUCUAGAGCAG |

**Table S2: The sequence of shSNHG6**

|  | 5’ | stem | loop | stem | 3’ |
| --- | --- | --- | --- | --- | --- |
| shSNHG6 -a | Ccgg | CGGCATGTATTGAGCATATAGGTTG | CTCGAG | CAACCTATATGCTCAATACATGCCG | TTTTTg |
| shSNHG6 -b | aattcaaaaa | CGGCATGTATTGAGCATATAGGTTG | CTCGAG | CAACCTATATGCTCAATACATGCCG |  |

**Table S3: The sequences of primers**

| Primer names | Sequences (5’-3’) |
| --- | --- |
| SNHG6 | Forward ATACTTCTGCTTCGTTACCT |
|  | Reverse CTCATTTTCATCATTTGCT |
| EZH2 | Forward GTACACGGGGATAGAGAATGTGG |
|  | Reverse GGTGGGCGGCTTTCTTTATCA |
| p15 | Forward GGACTAGTGGAGAAGGTGCG |
|  | Reverse GGGCGCTGCCCATCATCATG |
| p16 | Forward ATGGAGCCTTCGGCTGACT |
|  | Reverse GTAACTATTCGGTGCGTTGGG |
| p21 | Forward CGATGGAACTTCGACTTTGTCA |
|  | Reverse GCACAAGGGTACAAGACAGTG |
| p27 | Forward AACGTGCGAGTGTCTAACGG |
|  | Reverse CCCTCTAGGGGTTTGTGATTCT |
| p57 | Forward CTAGCCAGCAGGCATCGAG |
|  | Reverse GTGGTGGACTCTTCTGCGTC |
| CHIP Primer for p27 | Forward GGAAACATCTGCAGGCAACC |
|  | Reverse TGGTTCTCACTGGTTCAGGG |
| GAPDH | Forward GAAGTGTGAAGGTCGGAGTC |
|  | Reverse GAAGATGGTGATGGGATTTC |
